# Protocol of a randomized, double-blind, placebo-controlled, parallel-group, multicentre study of the efficacy and safety of nicotinamide in patients with Friedreich ataxia (NICOFA)

**DOI:** 10.1186/s42466-019-0038-9

**Published:** 2019-10-15

**Authors:** Kathrin Reetz, Ralf-Dieter Hilgers, Susanne Isfort, Marc Dohmen, Claire Didszun, Kathrin Fedosov, Jennifer Kistermann, Caterina Mariotti, Alexandra Durr, Sylvia Boesch, Thomas Klopstock, Francisco Javier Rodríguez de Rivera Garrido, Ludger Schöls, Thomas Klockgether, Massimo Pandolfo, Rudolf Korinthenberg, Philip Lavin, Geert Molenberghs, Vincenzo Libri, Paola Giunti, Richard Festenstein, Jörg B. Schulz, the EFACTS or NICOFA study group, Wolfgang Nachbauer, Andreas Eigentler, Elisabetta Indelicato, Matthias Amprosi, Perrine Charles, Claire Ewenczyk, Anna Heinzmann, Florian Holtbernd, Ilaria A. Giordano, Okka Kimmich, Florentine Radelfahr, Claudia Stendel, Stefanie Hayer, Mario Fichera, Lorenzo Nanetti, Anna Castaldo, Javier Arpa, Irene Sanz-Gallego, Daphne Babalis, Margarita Durkina

**Affiliations:** 1 Department of Neurology, RWTH Aachen University, Pauwelsstraße 30, 52074 Aachen, Germany; 2 JARA-BRAIN Institute Molecular Neuroscience and Neuroimaging, Forschungszentrum Jülich GmbH and RWTH Aachen University, 52074 Aachen, Germany; 3 Department of Medical Statistics, RWTH Aachen University, Pauwelsstraße 19, Aachen, Germany; 4 Center for Translational & Clinical Research Aachen (CTC-A), RWTH Aachen University, Pauwelsstraße 30, Aachen, Germany; 5 Unit of Genetics of Neurodegenerative and Metabolic Diseases, Fondazione IRCCS Istituto Neurologico Carlo Besta, Milan, Italy; 6 Genetic Department, ICM (Brain and Spine Institute) Sorbonne Universités, UPMC University Paris 06 UMR S 1127, and INSERM U 1127, CNRS UMR 7225 and APHP, Pitié-Salpêtrière University Hospital, Paris, France; 7 Department of Neurology, Medical University Innsbruck, Innsbruck, Austria; 8 Department of Neurology, Friedrich Baur Institute, University Hospital of the Ludwig-Maximilians-Universität Munich, Munich, Germany; 9 German Center for Neurodegenerative Diseases (DZNE), Munich, Germany; 10 Munich Cluster for Systems Neurology (SyNergy), Munich, Germany; 11 Reference Unit of Hereditary Ataxias and Paraplegias, Department of Neurology, IdiPAZ, Hospital Universitario La Paz, Madrid, Spain; 12 Department of Neurodegenerative Diseases, Hertie-Institute for Clinical Brain Research, University of Tübingen, Tübingen, Germany; 13 German Center for Neurodegenerative Diseases (DZNE), Tübingen, Germany; 14 Department of Neurology, University Hospital of Bonn, Bonn, Germany; 15 German Center for Neurodegenerative Diseases (DZNE), Bonn, Germany; 16 Laboratory of Experimental Neurology, Université Libre de Bruxelles, Brussels, Belgium; 17 Ethical Commission, Albert-Ludwigs-University Freiburg, Engelbergstr. 21, 79106 Freiburg, Germany; 18 Boston Biostatistics Research Foundation, Framingham, MA USA; 19 Interuniversity Institute for Biostatistics and Statistical Bioinformatics, UHasselt and KU Leuven, Leuven, Belgium; 20 NIHR UCLH Clinical Research Facility-Leonard Wolfson Experimental Neurology Centre, University College London (UCL) Institute of Neurology, London, UK; 21 Department of Molecular Neuroscience, University College London (UCL) Institute of Neurology, London, UK; 22 Gene Control Mechanisms and Disease Group, Department of Medicine, Division of Brain Sciences and MRC London Institute for Medical Sciences, Imperial College London, Hammersmith Hospital, London, UK

**Keywords:** Clinical study design, Clinical trials, Ataxia, Outcome, Frataxin

## Abstract

**Introduction:**

Currently, no treatment that delays with the progression of Friedreich ataxia is available. In the majority of patients Friedreich ataxia is caused by homozygous pathological expansion of GAA repeats in the first intron of the FXN gene. Nicotinamide acts as a histone deacetylase inhibitor. Dose escalation studies have shown, that short term treatment with dosages of up to 4 g/day increase the expression of FXN mRNA and frataxin protein up to the levels of asymptomatic heterozygous gene carriers. The long-term effects and the effects on clinical endpoints, activities of daily living and quality of life are unknown.

**Methods:**

The aim of the NICOFA study is to investigate the efficacy and safety of nicotinamide for the treatment of Friedreich ataxia over 24 months. An open-label dose adjustment wash-in period with nicotinamide (phase A: weeks 1–4) to the individually highest tolerated dose of 2–4 g nicotinamide/day will be followed by a 2 (nicotinamide group): 1 (placebo group) randomization (phase B: weeks 5–104). In the nicotinamide group, patients will continue with their individually highest tolerated dose between 2 and 4 g/d per os once daily and the placebo group patients will be receiving matching placebo. Safety assessments will consist of monitoring and recording of all adverse events and serious adverse events, regular monitoring of haematology, blood chemistry and urine values, regular measurement of vital signs and the performance of physical examinations including cardiological signs. The primary outcome is the change in the Scale for the Assessment and Rating of Ataxia (SARA) over time as compared with placebo in patients with Friedreich ataxia based on the linear mixed effect model (LMEM) model. Secondary endpoints are measures of quality of life, functional motor and cognitive measures, clinician’s and patient’s global impression-change scales as well as the up-regulation of the frataxin protein level, safety and survival/death.

**Perspective:**

The NICOFA study represents one of the first attempts to assess the clinical efficacy of an epigenetic therapeutic intervention for this disease and will provide evidence of possible disease modifying effects of nicotinamide treatment in patients with Friedreich ataxia.

**Trial registration:**

EudraCT-No.: 2017-002163-17, ClinicalTrials.gov NCT03761511.

## Introduction

### Background and rationale

#### Clinical trials in rare diseases

Preparing and carrying out investigator-initiated trials for rare diseases – in the hopes of developing new forms of therapy – is an even more difficult task than it is already with more prevalent diseases. Particularly with respect to funding, regulatory procedures as well as statistical planning and analyses, the demands are complex and require considerable additional and detailed knowledge. Trial designs for rare diseases must meet the same rigorous standards as those for trial for more prevalent diseases. A key question is how to run conclusive therapeutic trials. Patient groups are very small, and the risk of being underpowered to test efficacy is high. The recruitment of patients is a difficult task in rare diseases because of the paucity of available patients who fulfill the selection criteria, the quite heterogeneity of clinical presentation and/or co-morbidities [1], and finally because patients and/or their families are in continuous search for better treatments. Rare neurodegenerative diseases are fatal and no therapy is available to cure or slow down the progression of disease. This includes inherited ataxias. Within the European Friedreich’s Ataxia Consortium for Translational Studies (EFACTS, www.e-facts.eu), we started to address the challenge designing an investigator-initiated trial for patients with Friedreich Ataxia (FA) using an innovative trial methodology for design and analysis for patients with FA.

#### The disease – Friedreich ataxia

FA is an autosomal recessive hereditary ataxia. This devastating and incurable neurodegenerative disease often manifests in childhood and in many cases leads to severe disability by early adulthood. FA is the most common hereditary ataxia in Caucasian populations, however, with an estimated prevalence of about 2–4/100.000 people and a carrier frequency of 1:90 it remains a rare disease [2]. In 1996, the genetic mutation that underlies most FA cases [3] was discovered as a homozygous pathological expansion of GAA triplet repeats in the first intron of the *FXN* gene, which encodes for the mitochondrial protein frataxin. Frataxin deficiency results in spinocerebellar ataxia, dysarthria, proximal weakness, sensory loss and cardiomyopathy, and leads to dependence on a wheelchair and reduced life expectancy. There is no disease modifying therapy and many patients die prematurely of cardiomyopathy.

#### Study rationale and clinical relevance

It has been demonstrated that GAA-triplet repeats can trigger abnormal compaction of a linked reporter gene in vivo leading to the archetypal epigenetic gene silencing phenomenon of position effect variegation (PEV) [4]. This GAA-repeat silencing was highly sensitive to the gene dosage of PEV modifiers which encode enzymes that modify chromatin. It was subsequently found that the FXN gene is silenced at the chromatin level by the formation of heterochromatin and that this heterochromatin formation can be antagonised by histone deacetylase inhibitors (HDACi) [5].

Before the phase IIa trial [6] with nicotinamide was initiated in patients with FA, drug-specific preclinical studies were performed. First, nicotinamide was tested on EBV-transformed lymphoblastoid cell lines [5]. Modest up-regulation of *FXN* mRNA levels was obtained in patient-derived cell lines with relatively little effect on *FXN* expression in the normal control cell line. Second, to assess the effect of nicotinamide under more physiological conditions, fresh primary lymphocytes derived from FA-affected or normal healthy individuals were treated. A small effect (2-fold increase) in *FXN* mRNA level was produced for healthy primary cells after nicotinamide treatment suggesting that minor chromatin effects might occur at the healthy *FXN* locus. Notably, the increase in *FXN* mRNA level in FA individuals was much larger (4.5-fold). Third, in FA transgenic mice [7] treated with nicotinamide for 6 days with a dose of 750 mg/kg increases of *FXN* mRNA expression were found in all FA - relevant tissues and the most substantial increases, were observed in spinal cord and cerebellum with 1.8-fold up-regulation of frataxin [5]. These results strongly suggest that nicotinamide can reactivate the silenced *FXN* gene in both human FA cells and in vivo transgenic FA models. Also, mechanistic preclinical investigations were performed. Treating primary lymphocytes from FA patients with nicotinamide strongly reduced the disease-associated increase in histone H3K9 and H3K27 trimethylations and increased the histone acetylation at the *FXN* gene. The DNase I accessibility at the *FXN* gene was increased in FA patient’s cells. High-throughput RNA sequencing on libraries generated from untreated and nicotinamide-treated FA primary lymphocytes revealed that 67% of the dysregulation of the expression was ameliorated by the treatment in FA. Thus, it was demonstrated that expanded GAA-triplet repeats can induce gene silencing in vivo leading to the archetypal epigenetic gene silencing phenomenon of position effect variegation (PEV) [4]. This GAA-repeat silencing was exquisitely sensitive to the gene dosage of PEV modifiers which encode enzymes that modify chromatin. Moreover, it was demonstrated that the *FXN* gene is silenced at the chromatin level by the formation of heterochromatin. This heterochromatin formation can be antagonised by histone deacetylase inhibitors (HDACi) [5]. Nicotinamide is a classical class III HDACi.

A recent exploratory, open-label dose-escalation study on ten patients with FA demonstrated that frataxin levels can be restored towards asymptomatic carrier levels (50% of normal) using nicotinamide (ClinicalTrials.gov, number NCT01589809, 6]. Therefore, restoration of frataxin expression to these levels might be expected to halt disease progression. FA patients were given single doses, repeated daily doses of 2–8 g oral nicotinamide for 5 days and 8 weeks. Nicotinamide was generally well tolerated with nausea being the most frequent dose-related adverse event in this study. Moreover, a sensitive clinical rating measure to assess disease progression, the Scale for the Assessment and Rating of Ataxia (SARA) is now available, which was proved most suitable to measure clinical disease progression within the registry of the EFACTS framework [8, 9].

#### The drug – nicotinamide

Nicotinamide is a classical class III HDAC inhibitor [10], has a good bioavailability and rapidly penetrates all tissues, and readily crosses the blood brain barrier [11, 12]. Over the past 40 years nicotinamide has been given at high doses for a variety of therapeutic applications. A review on safety of high-dose nicotinamide has revealed that the therapeutic index of nicotinamide is wide but at very high doses reversible hepatotoxicity has been reported in animals and humans [13]. Abnormalities of liver enzymes can infrequently occur at the high or mega doses. There is no evidence of teratogenicity from animal studies and nicotinamide is not in itself oncogenic; at very high doses it does however potentiate islet tumor formation in rats treated with streptozotocin or alloxan. There is no evidence of oncogenicity in man. Growth inhibition can occur in rats but growth in children is unaffected. High-dose nicotinamide should still, however, be considered as a drug with toxic potential at adult doses in excess of 3 g/day and only applied under supervision. To our knowledge nicotinamide has been applied in FA in such high doses (> 3 g) only in the exploratory, open-label, dose-escalation study in FA in the UK [6]. Here, nicotinamide was given single doses, repeated daily doses of 2–8 g oral nicotinamide for 5 days and 8 weeks. Nicotinamide was generally well tolerated with nausea being the most frequent dose-related adverse event in this study; this was readily controlled with the use of antiemetics (typically domperidone up to four times daily or metoclopramide 10–20 mg IV PRN up to three times daily, either prophylactically prior to dosing and post-dosing if required) and by dose modification. Here, nicotinamide was rapidly absorbed after oral administration and generally well tolerated in FA patients after repeated daily dosing for 8 weeks. The following most common adverse events have been described in the exploratory, open-label, dose-escalation study by [6]: Nausea, headache, lightheadedness, vomiting, hypersomnia, fatigue, diarrhea, raised aspartate transaminase (AST) and alanine transaminase (ALT), falls, anorexia, migraine, dizziness, sore throat, retching, cold, fever, infections, cough, anemia and flu-like symptoms.

Moreover, nicotinamide was given safely for 5 years at about 3 g (1.2 g/m^2^) per day to more than 250 individuals including children from 3 years and above, adolescents and adults in an attempt to prevent diabetes in an at-risk population [14]. No serious adverse effects were observed.

## Methods

### Trial design

This is a prospective, multicentre, double-blind, randomized, placebo-controlled, phase IIb/III withdrawal trial, with a two-arm parallel group design to test the superiority hypothesis of nicotinamide in patients with FA (NICOFA) using an intended 2:1 allocation ratio. The allocation ratio is 2:1, favouring the active treatment to make the trial more attractive to patients with FA.

### Study setting

**Fig. 1:**
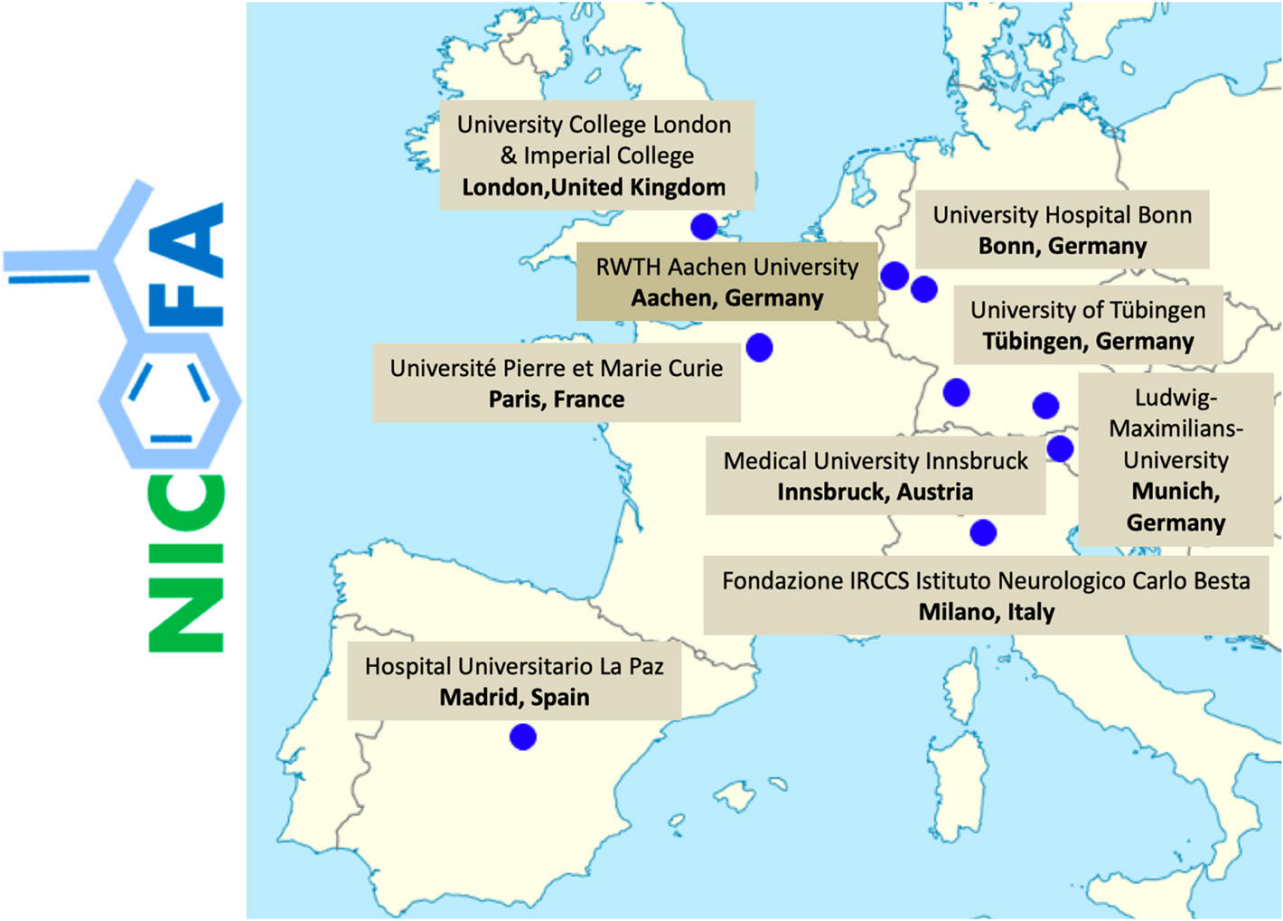
NICOFA centres

NICOFA will be performed at 10 academic hospital centres across Europe (Innsbruck, Austria; Paris, France; Milano, Italy; twice in London, United Kingdom; Madrid, Spain; Aachen, Bonn, Munich, and Tübingen in Germany), please see Fig. 1. Further details of the participating study sites can be obtained at ClinicalTrials.gov NCT03761511.

### Eligibility criteria

Key inclusion criteria are the molecular genetic diagnosis of FA with a GAA-repeat expansion on both alleles of the FXN gene and a SARA Score > 7 and < 28 and age ≥ 18 and < 50 years (Table 1). These inclusion criteria were chosen based on natural history data of disease progression [8, 9].

**Table 1:** Inclusion and exclusion criteria

Inclusion Criteria	
1. Patients must have a molecular genetic diagnosis of Friedreich ataxia with a GAA-repeat expansion on both alleles of the FXN gene and a SARA Score > 7 and < 28.	
2. Patients must be ≥18 years and < 50 old and have a weight of at least 50 kg.	
3. Written informed consent prior to study participation	
4. A female subject is eligible to participate if she is of: Non-childbearing potential defined as pre-menopausal females with a documented tubal ligation or hysterectomy; or postmenopausal defined as 12 months of spontaneous amenorrhea or of childbearing potential and agrees to use of highly effective birth control methods (Pearl Index < 1).	
Exclusion Criteria	
1. Patients with any medical condition or illness that, in the opinion of the investigator would interfere with study compliance and/or impair the patient’s ability to participate or complete the study.	
2. Any uncontrolled medical or neurological/neurodegenerative condition (other than Friedreich ataxia).	
3. Clinically significant psychiatric illness (e.g., uncontrolled major depression, schizophrenia, bipolar affective disorder) within 6 months prior to screening.	
4. Patients with significant clinical dysphagia that will be screened with dysphagia screening questionnaire.	
5. Hypersensitivity to nicotinamide.	
6. Patients known to be positive for human immunodeficiency virus (HIV).	
7. Patients with a significant history of substance abuse (e.g. alcohol or drug abuse) within the previous 6 months before enrolment. Substance abuse refers to the harmful or hazardous use of psychoactive substances, including alcohol and illicit drugs.	
8. Patients with a history of severe allergies.	
9. Indication of impaired liver function as shown by an abnormal liver function profile at Screening (e.g., repeated values of aspartate aminotransferase [AST], alanine aminotransferase [ALT] and bilirubin ≥3 × the upper limit of normal).	
10. History of malignancy or carcinoma. The following exceptions may be made after discussion with the Sponsor:	
• Subjects with cancers in remission more than 5 years prior to screening.	
• Subjects with a history of excised or treated basal cell or squamous carcinoma.	
• Subjects with prostate cancer in situ.	
11. History or evidence of an autoimmune disorder considered clinically significant by the Investigator or requiring chronic use of systemic corticosteroids or other immunosuppressants.	
12. History of clinically significant cardiac disease (ejection fraction < 40% [normal range 50–70%], cardiac insufficiency defined as New York Heart Association [NYHA] Class > 2; clinically significant congenital or acquired valvular disease; symptomatic coronary disease such as prior myocardial infarction or angina, B-type natriuretic peptide (BNP) level increase more than 2 x of the normal age- and gender dependent range; history of unstable arrhythmias, history of atrial fibrillation).	
13. The subject received an investigational drug within 30 days prior to inclusion into this study.	
14. Patients taking sodium valproate, tranylcypromine (monoamine oxidase inhibitor [MAOI]) or any other known histone deacetylase inhibitor.	
15. Use of vitamin B1 (thiamine), withdrawal should be at least 3 months prior screening or 5 half-lives, whichever is longer.	
16. Use of vitamin B3 (nicotinamide), withdrawal should be at least 3 months prior screening.	
17. If patients are taking idebenone or coenzyme Q_10_ (CoQ), this should be stable over the last 3 months and not changed during the study.	
18. The subject is unwilling or unable to provide written informed consent and to follow the procedures outlined in the protocol.	
19. For subjects who will undergo an MRI: Any contraindications to MRI such as, but not limited to cardiac pacemaker, implanted cardiac defibrillator, aneurysm clips, carotid artery vascular clamp, neurostimulator, implanted drug infusion devices, metal fragments or foreign objects in the eyes, skin or body, bone growth/fusion stimulator, cochlear, otologic implant, sever claustrophobia or any condition that would counterindicate an MRI scan.	
20. Patients participating in another interventional clinical trial, excluding natural history/observational studies, at start of the study or within the last 30 days before study start.	
21. The subject is mentally or legally incapacitated.	
22. Pregnant females as determined by positive [serum or urine] hCG test at Screening or prior to dosing. Participants of child-bearing age should use adequate contraception as defined in the study protocol.	
23. Lactating females.	

### Interventions

**Fig. 2:**
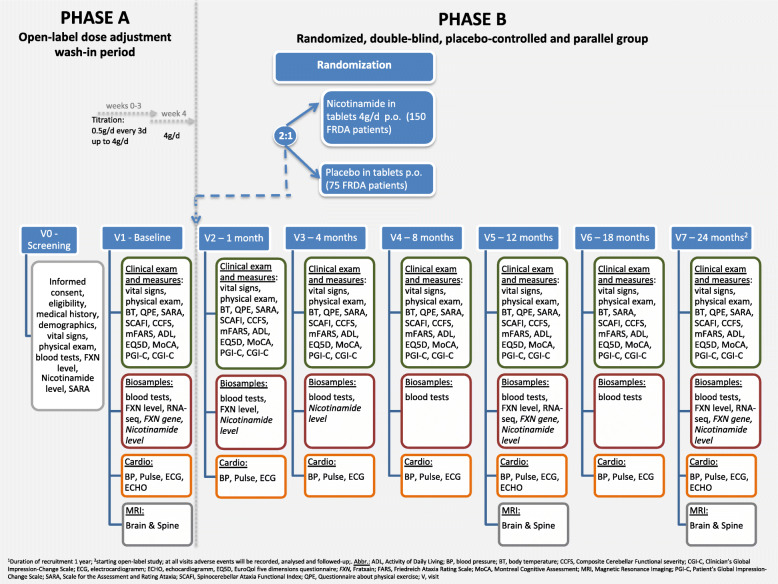
Study Flow Chart

Patients will undergo a 4 week, open-label, dose-adjustment period (*Phase A*), see also Fig. 2. This is an open-label dose adjustment wash-in period with nicotinamide: All patients will be slowly titrated with an increase of 0.5 g nicotinamide every 3 days up to a dose of 4 g/day. Thus, the titration period will be 3 weeks, patients should then be stable on 4 g/d or the highest tolerated dose for 1 week. Phase A is immediately followed by a 2:1 randomization for *Phase B* compromising the weeks 5–104. In *Phase B* there are two arms. The *nicotinamide* Group (2/3 of patients) receiving 4 g *nicotinamide* (capsules) or highest tolerated dose with a minimum of 2 g/d per os once daily and the Placebo Group (1/3 of patients) receiving matching Placebo (capsules) once daily. The expected duration of patient recruitment into the study will be 12 months; the study will be 24 months per patient and in total 36 months including evaluation and clinical study report.

Safety assessments will consist of monitoring and recording all adverse events and serious adverse events, the regular monitoring of blood and urine values, regular measurement of vital signs and the performance of physical examinations including cardiological signs.

Patients are allowed to continue participation in natural history studies but not in other clinical trials during participation in NICOFA.

### Outcomes

The primary objective of the study is to evaluate the efficacy of daily doses of nicotinamide in slowing disease progression as measured by changes in the SARA as compared with placebo in patients with FA. SARA is validated for FA and shown to be the most suitable measure of disease progression in FA [8, 9].

Secondary objectives are to determine the change of secondary endpoints such as quality of life, functional motor and cognitive measures, clinician’s and patient’s global impression-change scales as well as the up-regulation of the frataxin protein level, safety and survival/death. Secondary endpoints will include: Activity of Daily Living (ADL), part of the Friedreich ataxia rating scale (FARS), the EuroQol five dimensions questionnaire (EQ-5D); the modified Friedreich Ataxia Rating Scale (mFARS) [15], the Spinocerebellar Ataxia Functional Index (SCAFI) [16]; the Composite Cerebellar Functional Severity (CCFS), the up-regulation of frataxin protein level, the Clinician’s Global Impression-Change Scale (CGI-C); the Patient’s Global Impression-Change Scale (PGI-C), and safety and survival/death. Tertiary objectives are mainly cognitive (Montreal Cognitive Assessment (MoCA)), genetics, cardiological (echocardiogram), and neuroimaging measures of the central nervous system (spinal cord and brain magnetic resonance imaging (MRI)) [17, 18].

### Participant timeline

The study duration is 24 months per patient, in total 39 months including evaluation and clinical study report (Fig. 2, Table 2). The recruitment period will last 12 months, and data cleaning, processing, analysis and reporting about 3 months.

**Table 2:** Visit schedule

Study Period	Screening	Phase A 1 month	Phase B 23 months					
Visit No.		1	2	3	4	5	6	7
Activity		Baseline	Randomization 1 month	4 month	8 month	12 month	18 month	EOS 24 month
Visit window +/− calendar days			±3 days	±1 week	±1 week	±1 week	±1 week	±1 week
Informed consent	X							
Inclusion/Exclusion criteria^a^	X							
Medical history	X							
Pregnancy test	X	X	X	X	X	X	X	X
Demography	X	X						
Vital signs^b^, respiratory rate, resting pulse, height and weight	X	X	X	X	X	X	X	X
Blood pressure^b^	X	X	X	X	X	X	X	X
Body temperature	X	X	X	X	X	X	X	X
Physical examination	X	X	X	X	X	X	X	X
Electrocardiogram (ECG)		X	X	X	X	X	X	X
Echocardiogram (ECHO)		X				X		X
Quest. about exercise (QPE)		X	X	X	X	X	X	X
Clinical measures^c^ SCAFI, CCFS, mFARS, ADL, EQ. 5D, MoCA, CGI-C, PGI-C		X	X	X	X	X	X	X
Scale for the Assessment and Rating of Ataxia (SARA)	X	X	X	X	X	X	X	X
Standard laboratory evaluation^d^	X	X	X	X	X	X	X	X
Up-regulation of Frataxin (FXN) protein level		X	X			X		X
Gene expression of *Frataxin (FXN)* gene	X	X				X		X
RNA-Sequencing of *Frataxin* gene		X				X		X
PK sampling (nicotinamide level)	X	X	X	x		X		X
MRI (brain and spine)		X				X		X
Concomitant medication	X	X	X	X	X	X	X	X
Adverse events evaluation		X	X	X	X	X	X	X

^a^ might include genetic testing, if participants are not included in the EFACTS registry

^b^ after at least 15 min of resting

^c^ *ADL* Activity of daily living, *CCFS* Composite cerebellar functional severity, *CGI-C* Clinician’s global impression-change scale, *EQ. 5D* EuroQol five dimensions questionnaire, *INAS* Inventory of non-ataxia signs, *MoCA* Montreal cognitive assessment, *PGI-C* Patient’s global impression-change scale, *SCAFI* Spinocerebellar ataxia functional index, *EOS* End of Study

^d^ Hematology (total and differential blood count) and blood chemistry

### Sample size

Within the EFACTS register [8, 9], inclusion of patients with a SARA < 28 and an age < 50 years resulted in a slope i.e. the progression in SARA score of 1.19 per year; established by fitting a linear mixed effects model to the data. To determine a 50% reduction in the progression in the treatment group by a corresponding linear mixed effects model was used to test the difference in the slopes at the (two-sided) 5% significance level with a two-year observational period and scheduled visit at baseline and month 4, 8, 12, 18 and 24, a sample size of 150 + 75 = 225 is required (90% power, 2:1 allocation ratio).

Within the range of observed SARA measurements, it has been shown, that SARA progression is mainly linear over time in a cohort that fulfills the inclusion criteria [8, 9].

### Recruitment

For enrolment of the necessary total sample size of 225 patients screening of 360 patients is expected to be necessary. Patients will be recruited via EFACTS, ataxia outpatient centres and patient advocacies.

### Randomization and blinding

The randomization list prepared by the Department of Medical Statistics of the RWTH Aachen University Hospital, Aachen in Germany using randomizeR is stratified by center. The best practice randomization procedure to minimize the impact of selection and time trend bias on the type one error will be selected via a simulation study (ERDO) [19]. The packaging of the investigational product and placebo following the randomization list will be done in Mainz, Germany, labelled with a randomisation code. This will maintain concealment and double blinded treatment allocation. After randomization neither the patients nor the investigator or sponsor will be aware of the treatment allocation. Patients assigned to one of the double-blinded treatments will take nicotinamide capsules or matching placebo. The capsules will be identical in appearance. The involved staff of the Department of Medical Statistics of the RWTH Aachen University Hospital as well as the involved staff of the pharmacy are aware of the randomized treatment allocation sequence but keep the list and information concealed till closure of the database. Neither the Department of Medical Statistics of the RWTH Aachen University Hospital nor the pharmacy are involved in patient recruitment.

### Statistical analysis

Analyses will be performed within the Full Analysis Set (FAS), which is the set of subjects who have been randomized and have taken at least one dose of the trial medication. Evaluation of the primary endpoint SARA score is based on the linear mixed effect model (LMEM) with the fixed effects of treatment, treatment-by-time interaction, center, SARA baseline score and age as well as the random intercept and time. Treatment effect is descripted by 95% confidence intervals based on the contrast of the treatment progression. Similar models will be used to test the treatment effects for the other outcomes.

Taking into account the concept paper on an addendum to ICH E9 statistical principles for clinical trials, we will further model estimates by an advanced sensitivity analysis, i.e. fitting a an ignorable likelihood model to the data [20]. Such model will be fitted using the method described in Mallinckrodt [20] with the fixed effects treatment, time and treatment by time interaction as well as the covariates baseline SARA score and the baseline-by-visit interaction age of onset, age will be fitted to the data.

## Perspective

This clinical trial NICOFA will assess the clinical efficacy of an epigenetic therapeutic intervention for FA and provide evidence of possible disease modifying effects of nicotinamide treatment in patients with FA.

## Data Availability

At individual level and adhering to the General Data Protection Regulation, every patient has the right to get access to their personal data. Data are available upon request.
